# Unique fiber phenotype composition and metabolic properties of the stapedius and tensor tympani muscles in the human middle ear

**DOI:** 10.1111/joa.13861

**Published:** 2023-03-13

**Authors:** Anton Rönnblom, Lars‐Eric Thornell, Farhan Shah, Krister Tano, Per Stål

**Affiliations:** ^1^ Department of Clinical Science, Otorhinolaryngology/Sunderby Research Unit Umeå University Umeå Sweden; ^2^ Department of Integrative Medical Biology, Laboratory of Muscle Biology Umeå University Umeå Sweden

**Keywords:** capillaries, fiber type, middle ear, mitochondria, myosin heavy chain

## Abstract

The middle ear muscles have vital roles, yet their precise function in hearing and protection remains unclear. To better understand the function of these muscles in humans, the morphology, fiber composition, and metabolic properties of nine tensor tympani and eight stapedius muscles were analyzed with immunohistochemical, enzyme‐histochemical, biochemical, and morphometric techniques. Human orofacial, jaw, extraocular, and limb muscles were used as references. The immunohistochemical analysis showed that the stapedius and tensor tympani muscles were markedly dominated by fibers expressing fast contracting myosin heavy chain MyHC‐2A and MyHC‐2X (79 ± 6% vs. 86 ± 9%, respectively, *p* = 0.04). In fact, the middle ear muscles had one of the highest proportions of MyHC‐2 fibers ever reported for human muscles. Interestingly, the biochemical analysis revealed a MyHC isoform of unknown identity in both the stapedius and tensor tympani muscles. Muscle fibers containing two or more MyHC isoforms were relatively frequently observed in both muscles. A proportion of these hybrid fibers expressed a developmental MyHC isoform that is normally absent in adult human limb muscles. The middle ear muscles differed from orofacial, jaw, and limb muscles by having significantly smaller fibers (220 vs. 360 μm^2^, respectively) and significantly higher variability in fiber size, capillarization per fiber area, mitochondrial oxidative activity, and density of nerve fascicles. Muscle spindles were observed in the tensor tympani muscle but not in the stapedius muscle. We conclude that the middle ear muscles have a highly specialized muscle morphology, fiber composition, and metabolic properties that generally showed more similarities to orofacial than jaw and limb muscles. Although the muscle fiber characteristics in the tensor tympani and stapedius muscles suggest a capacity for fast, fine‐tuned, and sustainable contractions, their difference in proprioceptive control reflects different functions in hearing and protection of the inner ear.

## Graphical abstract

Comparison between the stapedius muscle of the middle ear (a) and a facial muscle, the zygomaticus major (b), a jaw muscle, the masseter (c) and a limb muscle, the biceps brachii (d). Note, the small fiber size and the high proportion of fast contracting type 2 fibers (stained red) in the middle ear and facial muscle compared to the jaw and limb muscle.

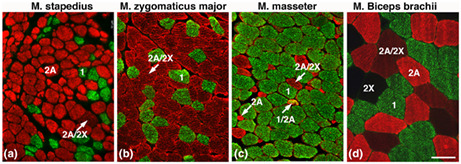

## 1 INTRODUCTION

In the middle ear, the transmission of sound vibrations and pressure changes to the cochlea is modulated by two muscles, the tensor tympani muscle, which is attached to the malleus shaft, and the stapedius muscle, which is attached to the stapes. Activation of the stapedius muscle, the smallest muscle in the human body, decreases the movements of the stapes in the oval window of the cochlea, while activation of the tensor tympani muscle pulls the malleus medially, increasing the tension of the tympanic membrane. In several mammals, both the stapedius and the tensor tympani muscles contract in response to auditory stimuli (Borg, 1972; Eliasson & Gisselsson, 1955; Murata et al., 1986), while in humans, the stapedius muscle reflex is considered to be the dominant acoustically evoked reflex pathway to protect the inner ear from loud noises (Mukerji et al., 2010). The reflex is triggered by loud sound (70–100 dB), which causes muscle contraction dampening the vibration of the stapes and decreasing the intensity of sound transmitted to the cochlea by about 15 dB. In contrast, the human tensor tympani muscle seems not to be activated by external sound but rather by tactile stimulation of facial areas, swallowing, phonation, and most commonly, as part of the startle reaction (Bance et al., 2013). It has also been attributed to regulating pressure in the middle ear (Sadé & Ar, 1997). Although various roles have been assigned to the stapedius and the tensor tympani muscle, the functions of these muscles in humans are still largely unclear and often speculative (Mukerji et al., 2010).

In muscles, the myosin heavy chain (MyHC) is the major component of the myosin motor protein which converts chemical energy into mechanical force. This motor protein exists in different isoforms, and the type of isoforms in a muscle determines its contractile properties. In humans, at least eight well‐defined MyHC isoforms are present, each encoded by a specific gene (Schiaffino & Reggiani, 2011). Three of these are identified in adult human limb muscles, one slow myosin isoform, MyHC‐1 (I/β), coded by the MyH7 gene, and two fast isoforms, MyHC‐2A and MyHC‐2X, coded by the MyH2 and MyH1 gene, respectively. Muscle fibers containing slow MyHC‐1 or fast MyHC‐2A have a relatively higher mitochondrial oxidative capacity and capillary supply than MyHC‐2X fibers, which, on the other hand, have higher contraction velocity and power during short activities (Schiaffino & Reggiani, 2011). A high proportion of muscle fibers co‐expressing mixtures of different MyHC isoforms, that is, hybrid fibers, give the muscles a wide range of physiological properties.

Muscles in the craniofacial region are structurally and functionally more specialized than limb muscles (Stål et al., 1987, 1990, 1994, 1996, 2003; Stål & Lindman, 2000), where some contain additional MyHC isoforms that are not normally present in adult limb muscles. Adult human jaw, orofacial, and extraocular muscles (EOMs) express two developmental MyHC isoforms, MyHC‐emb coded by the MYH3 gene, and MyHC‐neo coded by the MyH8 gene (Ciciliot & Schiaffino, 2010; Sartore et al., 1987; Stål et al., 1994; Stål & Lindman, 2000). Developmental MyHCs normally disappear in limb muscles shortly after birth when adult fast and slow myosin become prevalent (Ciciliot & Schiaffino, 2010). Moreover, human jaw muscles also differ from limb muscles by expressing an α‐cardiac MyHC isoform (MyHC‐α, gene MyH6), and human EOMs contain a specific fast MyHC‐EO isoform (gene MyH13) and a MyHC‐slow tonic isoform (gene MyH7b). Moreover, small mammals also have a third fast MyHC isoform, MyHC‐2B, coded by the MyH4 gene, which has a rapid myosin cross‐bridge cycle. The transcript for MYH‐2B has previously been identified in humans (Horton et al., 2001; Smerdu & Cvetko, 2013), but the corresponding MyHC‐2B protein has not been demonstrated in human muscles.

Despite the vital roles of the middle ear muscles in protecting the inner ear, the morphology and physiological properties of these muscles, such as power, speed of shortening, and fatigue resistance, are largely unknown. To better understand the functions of the human middle ear muscles, this study aimed to investigate the muscle morphology, MyHC composition, oxidative capacity, and vascularization of the human stapedius and tensor tympani muscles.

## 2 MATERIALS AND METHODS

### 2.1 Approval of the study

Autopsy collection was done according to Swedish laws and regulations on autopsy and transplantation and in agreement with the declaration of Helsinki. Consent was given by the Swedish National Board of Health and Welfare (Dnr 5254–17,784).

### 2.2 Muscle samples

The middle part of almost the entire musculus tensor tympani (five females, four males, mean age: 59.8, range: 28–95 years) and musculus stapedius (three females, five males, mean age: 68.4, range: 28–95 years) were obtained postmortem from previously healthy subjects. For comparison, muscle samples were obtained from the orofacial, jaw, and limb muscles of five previously healthy subjects (range: 15–75 years). The orofacial samples were taken from the middle part of the zygomaticus major and the palatopharyngeus muscle (three subjects). The jaw muscle sample was taken from the middle superficial part of the anterior masseter muscle, and the samples from the limb were obtained from the middle superior parts of both the biceps brachii (long head) and the vastus lateralis. For analysis of MyHC isoforms by gel electrophoresis, one additional muscle, the oblique superior extraocular muscle (EOM), was used. All muscle specimens were obtained 1–2 days postmortem, a delay acceptable for obtaining reliable fiber typing (Eriksson et al., 1980). The samples were mounted for transverse sectioning in optimal cutting temperature (OCT) compound (Tissue‐Tek; Miles Inc.), rapidly frozen in liquid propane, chilled with liquid nitrogen, and stored at −80°C until further processing.

### 2.3 Immunohistochemistry

Serial muscle cross‐sections, 5–6 μm thick, were cut in a cryostat at −20°C and mounted on glass slides. Immunohistochemical staining was performed using well‐characterized monoclonal antibodies (mAbs) and modified standard immunohistochemical techniques. For antibody specificity and concentrations, see Table 1. In brief, the sections were immersed in 5% normal nonimmune donkey serum (Jackson ImmunoResearch Laboratories, Inc.) for 15 min and after that, rinsed in 0.01 M phosphate‐buffered saline (PBS) for 3 × 5 min. The sections were then incubated with the primary antibodies diluted to appropriate concentrations in PBS with bovine serum albumin (BSA) in a humid environment. Incubation was carried out overnight at 4°C. After additional washes in PBS, the sections were incubated with the secondary antibody (Ab) (37°C for 30 min) and washed in PBS for 3 × 5 min. Bound primary Abs were visualized by indirect immunofluorescence using affinity‐purified Abs prepared for multiple labeling and conjugated with fluorochrome with different emission spectra; fluorescein isothiocyanate (FITC), Rhodamine Red‐X (RRX; Jackson ImmunoResearch Laboratories, Inc.), Alexa Fluor 488 and Alexa Fluor 647 (Invitrogen). The sections were thereafter washed in PBS for 3 × 5 min and then mounted in Vectashield Mounting Medium (H‐1000; Vector Laboratories) or mounting medium with 4**′**,6‐diamidino‐2‐phenylindole (DAPI) for staining of nuclei (Vector Laboratories). For details, see Shah et al., 2016.

**TABLE 1:** Antibodies used for immunohistochemistry.

Antibodies	Specificity	Gene^a^	Dilution	Source identification
A4.951	MyHC‐1(beta)	*MYH7*	1:500	DSHB Cat# A4.951, RRID:AB_528385
A4.840	MyHC‐1	*MYH7*	1:30	DSHB Cat# A4.840, RRID:AB_528384
A4.74	MyHC‐2A	*MYH2*	1:400	DSHB Cat# A4.74, RRID:AB_528383
BF‐F3	MyHC‐2B	*MYH4*	1:100	DSHB Cat# BF‐F3, RRID:AB_2266724
N2.261	MyHC‐1	*MYH7*	1:50	DSHB Cat# N2.261, RRID:AB_531790
	MyHC‐2A	*MYH2*		
	MyHC‐EO	*MYH13*		
	MyHC‐α	*MYH6*		
S58	MyHC‐slow tonic	*MYH7b*	1:500	DSHB Cat# s58, RRID:AB_528377
F1.652	MyHC‐emb	*MYH3*	1:5	DSHB Cat# F1.652, RRID:AB_528358
NCL‐MHCn	MyHC‐neo	*MYH8*	1:200	Leica Biosystems Cat# NCL‐MHCn, RRID:AB_563900
F88 12F8	MYHC‐α	*MYH6*	1:5	Alexis Biochemicals (Enzo), San Diego, CA, USA
4C7	Laminin α5 chain	*LAMA5*	1:200	Agilent Cat# M0638, RRID:AB_2249754
5H2	Laminin α2 chain	*LAMA2*	1:1000	Leica Biosystems Cat# NCL‐MEROSIN, RRID:AB_442108
D33	Desmin	*DES*	1:1000	Agilent Cat# M0760, RRID:AB_2335684

*Note*: MAbs A4.951, A4.840, A4.74, BF‐F3, N2.261, S58, and F1.652 are obtained from The Developmental Studies Hybridoma Bank, developed under the auspices of the NICHD and maintained by The University of Iowa, Dept of Biological Sciences, Iowa City, IA, USA.

^a^ Official gene nomenclature according to OMIM. (http://www.ncbi.nlm.nih.gov/omim/).

### 2.4 Enzyme histochemistry

To demonstrate the oxidative capacity of the muscle fibers, the muscle cross‐sections were stained for mitochondrial enzyme nicotinamide adenine dinucleotide‐tetrazolium reductase (NADH‐TR) (EC 1.6.99.3) (Nyström, 1968).

### 2.5 Morphometric analysis

The entire or nearly the whole muscle cross‐section area in both the stapedius and tensor tympani muscle samples was scanned in immunohistochemically stained sections using a digital high‐speed fluorescence charge‐coupled device (CCD) camera (Leica DFC360 FX) connected to a fluorescence microscope (Leica DM6000B; both Leica Microsystems CMS GmbH). Calculation of the muscle fiber cross‐sectional area (CSA) and muscle fiber capillarization were performed with a custom morphometric software package (Leica QWin Standard version 3.5.1, Leica Microsystems Ltd.). The circumference (basal lamina) of each fiber and each capillary was traced on the computer image in sections stained for laminin α‐2 chain in fibers and α‐5 chain in capillaries. The measurements of fiber area and fiber type proportion were based on 7034 muscle fibers in the tensor tympani (a mean of 782 fibers per subject, range: 304–1433) and 3202 muscle fibers in the stapedius muscles (a mean of 400 fibers per subject, range: 169–503). In the orofacial, jaw, and limb muscles, the number of analyzed fibers was >200 fibers from each muscle sample. The quantification of capillarization of the middle ear muscles was limited to six tensor tympani and three stapedius muscles owing to a lack of muscle tissue. The analysis of muscle fiber capillarization was based on 2505 fibers in the tensor tympani muscle (a mean of 418 fibers per subject, range: 105–537) and 1808 fibers in the stapedius muscles (a mean of 363 fibers per subject, range: 255–497). In a normal human limb muscle, capillarization of 50 fibers is sufficient to make a robust analysis of the capillary network (McCall et al., 1998).

### 2.6 Muscle fiber classification and estimation of the proportion

Based on the immunostaining pattern for the different MyHC mAbs, the muscle fibers were classified as fibers expressing slow MyHC‐1, fast MyHC‐2A, or fast MyHC‐2X, or as hybrid MyHC‐1/2A or MyHC‐2A/2X fibers. The proportion (%) of different MyHC fiber types was calculated by dividing the number of different classified MyHC fibers by the total number of fibers in the cross‐sections.

### 2.7 Variability in muscle fiber diameter

Variability in fiber area was expressed for each fiber type as the coefficient of variation (CV). The CV was calculated according to the formula standard deviation x 1000/mean fiber area.

### 2.8 Capillary variables

Capillary density (CD) was calculated as the total number of capillaries per mm^2^ in the cross‐section of muscle fibers. To analyze the number of capillaries around each fiber (CAF), all capillaries within a distance of 5 μm from each individual muscle fiber were included. Capillaries related to each individual fiber cross‐sectional area (CAFA) were calculated according to the formula CAF/fiber cross‐sectional area × 10^3^.

### 2.9 Biochemical methods

Frozen muscle cross‐sections (10–40 μm thick) from five tensor tympani and five stapedius muscles, one EOM, and one biceps brachii muscle were placed in a Laemmli sample buffer (Bio‐Rad Laboratories AB). Myosin heavy chain isoforms were analyzed in 8% sodium dodecyl sulfate (SDS)‐glycerol gels containing 30% glycerol (Talmadge & Roy, 1993). Protein fractions were separated in the Bio‐Rad mini‐Protean 3‐cell system (Bio‐Rad Laboratories). The upper buffer contained 10 mM 2‐mercaptoethanol (Blough et al., 1996). The gels were silver‐stained for 24 h at 70 V and scanned in a soft laser densitometer (Molecular Analyst software; Bio‐Rad Laboratories) to determine the relative content of MyHC isoforms. For immunoblotting, the separated proteins were transferred to nitrocellulose sheets and exposed to mAbs A4.951 and A4.74.

### 2.10 Muscle spindles

Muscle spindles were in transverse muscle sections defined as groups of small‐sized muscle fibers surrounded by a connective tissue capsule.

### 2.11 Statistical analysis

All statistical tests were performed using SPSS version 23 (IBM SPSS Statistics; IBM Corporation). Normality in the distribution of data was tested using the Shapiro–Wilk test. Comparisons between more than two groups were made using one‐way ANOVA, and significance between individual groups was confirmed using the Tukey post hoc test. Differences between the two groups were made using the independent sample *t*‐test for variables with normal distribution and the Mann–Whitney *U* test for non‐normally distributed variables. *p* ≤ 0.05 was considered significant

## 3 RESULTS

### 3.1 General muscle morphology and immunoreaction of antibodies

**FIGURE 1:**
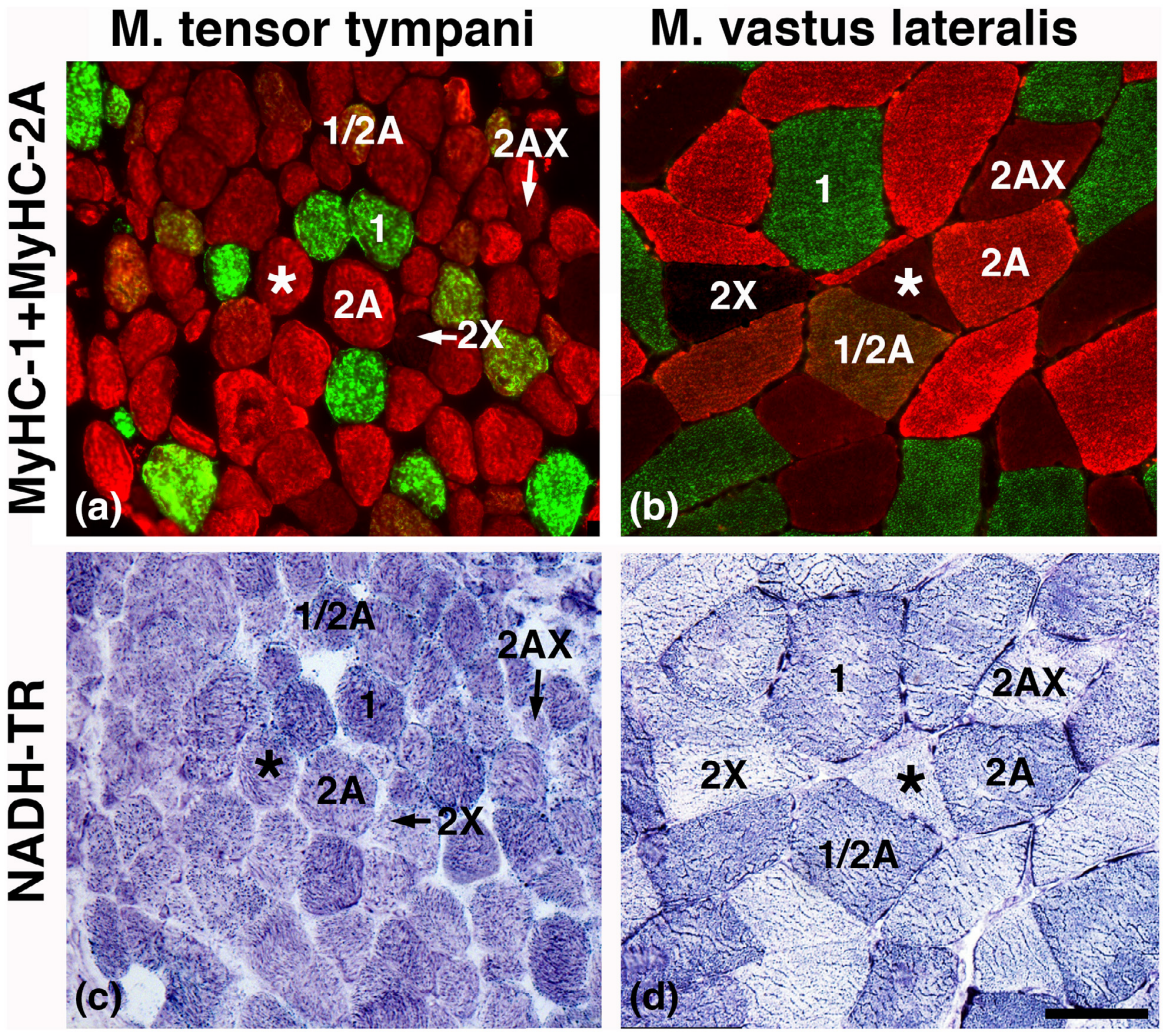
Muscle fiber phenotypes and mitochondrial activity in a middle ear muscle, the tensor tympani, and one limb muscle, the vastus lateralis. Cross‐sections a and b are double‐stained for MyHC‐1 (red) and MyHC‐2A (green). Muscle fibers co‐expressing MyHC‐1 and MyHC‐2A (1 + 2A) are stained brown, fibers co‐expressing MyHC‐2A and MyHC‐2X are stained light red, and fibers expressing MyHC‐2X are unstained. Panels c and d show MyHC fiber types in serial cross‐sections stained for NADH‐TR in corresponding muscle. Note the higher mitochondrial NADH‐TR activity of fibers in the tensor tympani muscle, especially in fibers expressing MyHC‐2 isoforms, compared with the vastus lateralis muscle. Scale bar 50 μm.

**FIGURE 2:**
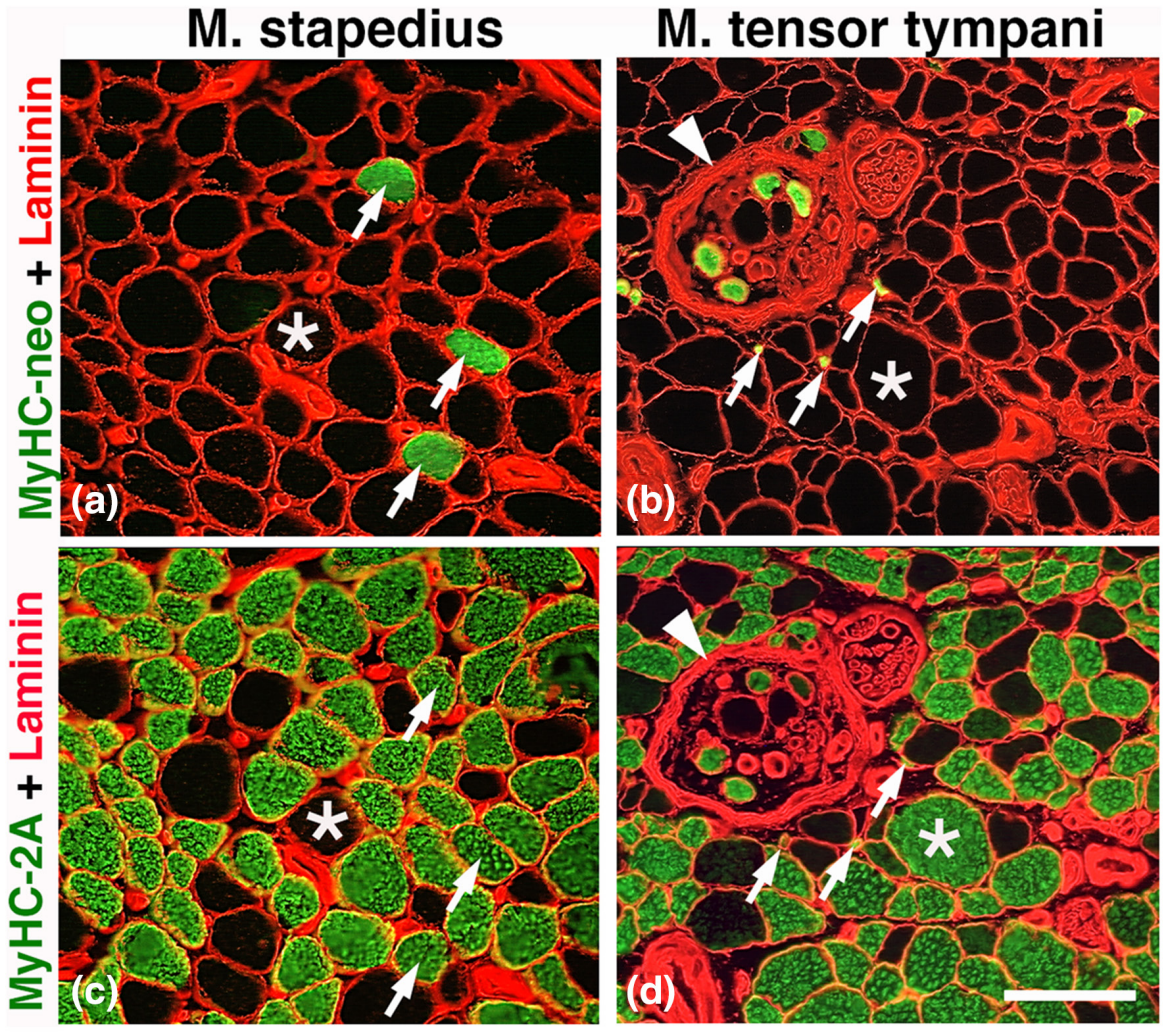
Expression of MyHC‐neo (a, b, arrows) in the stapedius (a, c) and a tensor tympani muscle (b, d). The cross‐sections are immunostained for laminin and MyHC‐neo (a and b) and laminin and MyHC‐2A (c and d). Asterix shows the corresponding fiber in each muscle. Note that MyHC‐neo is co‐expressed in MyHC‐2A fibers. Note also the presence of a muscle spindle in the tensor tympani muscle (b, d, arrowhead), where some intrafusal fibers are stained for both MyHC‐neo and MyHC‐2A (b and d). Scale bar 50 μm.

**FIGURE 3:**
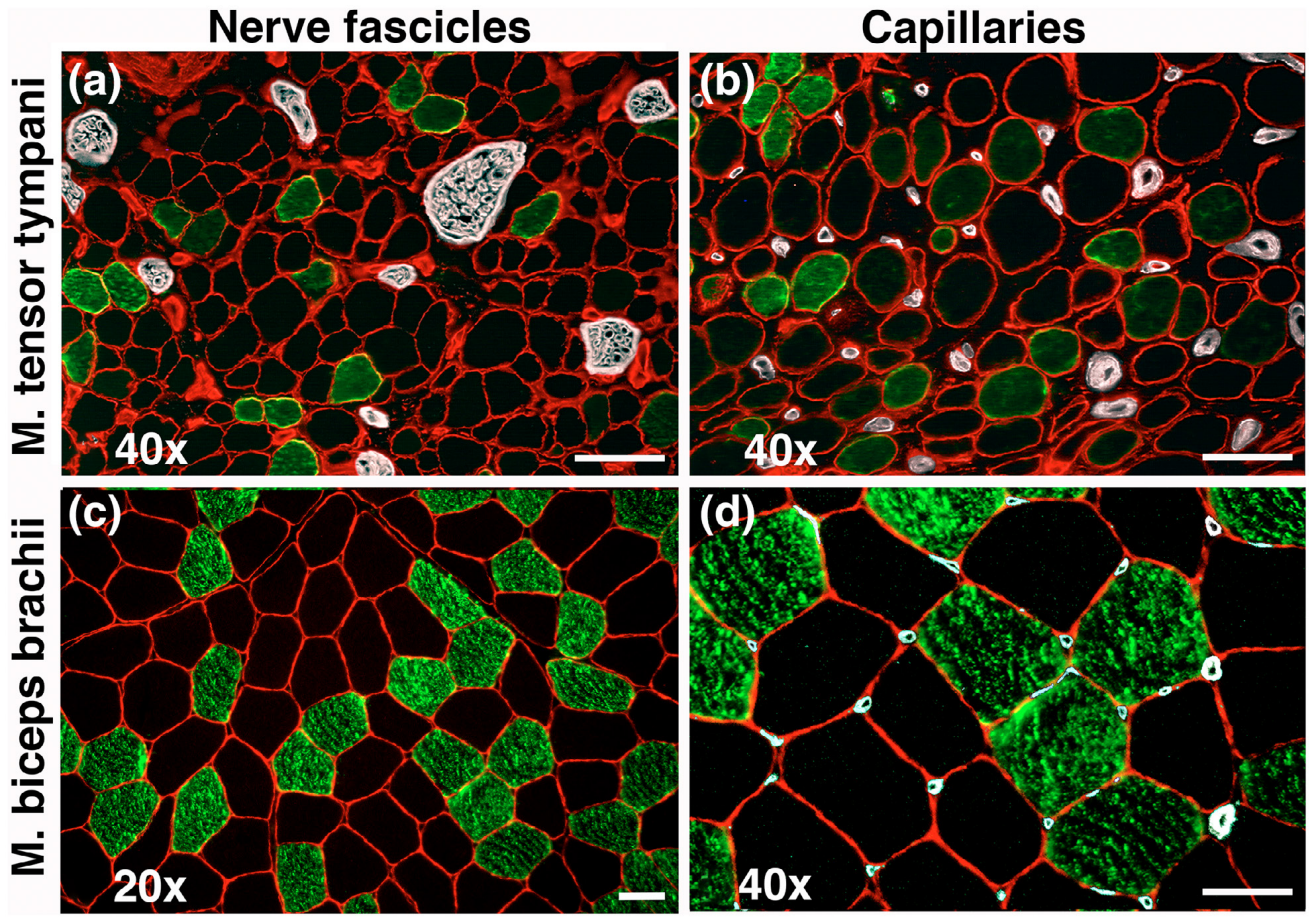
Cross‐sections from a middle ear muscle, the tensor tympani (a and b), and one limb muscle, the biceps brachii (b and d), showing the density of nerve fascicles (a and c) and capillaries (c and d). Panel c is scanned in a lower magnification (20×). The sections are immunostained for laminin in the basement membrane (red) and MyHC‐1 in fibers (green). Nerves and capillaries are marked white. Note the high density of nerve bundles in the tensor tympani muscle (a) compared with the lack of nerve fascicles in the area from the biceps brachii muscle (c). Note also the higher density of capillaries in the muscle cross‐sections of the tensor tympani muscle (b) compared to the limb muscle (d). Scale bars 50 μm.

The tensor tympani and the stapedius muscles were characterized by a population of relatively less densely packed small‐sized fibers, often with a round rather than a polygonal shape. However, the interindividual and intramuscular variability in fiber size and fiber form was noticeable in both muscles (Figure 1). Muscle spindles were observed in three of the tensor tympani muscle samples, while no muscle spindles were found in the stapedius muscles (Figure 2). The density of nerve bundles in the muscle cross‐section was observed to be higher in the tensor tympani and stapedius than in the limb muscles (Figure 3).

All middle ear muscles contained fibers expressing MyHC‐1, MyHC‐2A, and MyHC‐2X, and fibers containing a mixture of these MyHC isoforms. A low proportion of muscle fibers also co‐expressed MyHC‐neo in most samples (Figure 2). No extrafusal muscle fibers showed immunoreaction for MyHC‐2B, MyHC‐emb, MyHC‐slow tonic, or MyHC‐α.

### 3.2 Musculus stapedius

#### 3.2.1 Frequency of muscle fiber phenotypes

All muscle samples were predominantly composed of fibers expressing fast MyHC‐2 isoforms (mean 79 ± 6%), where MyHC‐2A was most frequent (mean: 70 ± 13%). Muscle fibers containing solely slow MyHC‐2X were only found in one subject, while fibers containing MyHC‐1 were present in all muscle samples (mean: 21 ± 6%). Hybrid fibers expressing MyHC‐1/2A and MyHC‐2A/2X were observed in five of the eight muscle samples (mean: 2 ± 4% and 6 ± 9%, respectively) (Table 2; Figure 1).

**TABLE 2:** Frequency (%) of fibers expressing different MyHC isoforms in the stapedius and the tensor tympani muscles.

Muscle	Cases (*n*)	MyHC‐1	MyHC‐1/2A	MyHC‐2A	MyHC2A/2X	MyHC‐2X
Middle ear muscles						
Stapedius	8	21 ± 6	2 ± 4	70 ± 13	6 ± 9	1 ± 1
Tensor tympani	9	14 ± 9	5 ± 6	74 ± 17	6 ± 10	1 ± 2
Cranial muscles						
Zygomaticus major	3	21 ± 1^†^	1 ± 0.2	16 ± 8^*†^	58 ± 10^*†^	4 ± 1^*†^
Palatopharyngeus	5	14 ± 16	2 ± 2	47 ± 58^†^	36 ± 25	2 ± 0.3
Masseter	5	54 ± 14^*†^	8 ± 7	0 ± 0^*†^	1 ± 2	37 ± 12^*†^
Limb muscles						
Biceps brachii	5	46 ± 7^*†^	2 ± 2	42 ± 7^*†^	7 ± 6	9 ± 10^*†^
Vastus lateralis	5	44 ± 8^*†^	5 ± 5	42 ± 11^*†^	8 ± 5	0 ± 0^†^

*Note*: For comparison, data from three cranial and two limb muscles are included. Data are presented as means ± standard deviations. Statistical differences (*p* < 0.05) to the stapedius muscle (*) and to the tensor tympani muscle (†) are marked.

#### 3.2.2 Frequency of fibers expressing neonatal myosin

Muscle fibers co‐expressing MyHC‐neo were found in six of the eight analyzed stapedius muscles (Figure 2). The average percentage of fibers containing MyHC‐neo in the stapedius muscle was 3.3 ± 3.2%. MyHC‐neo was not co‐localized with any particular MyHC fiber type.

#### 3.2.3 Muscle fiber area and variability in fiber size

**FIGURE 4:**
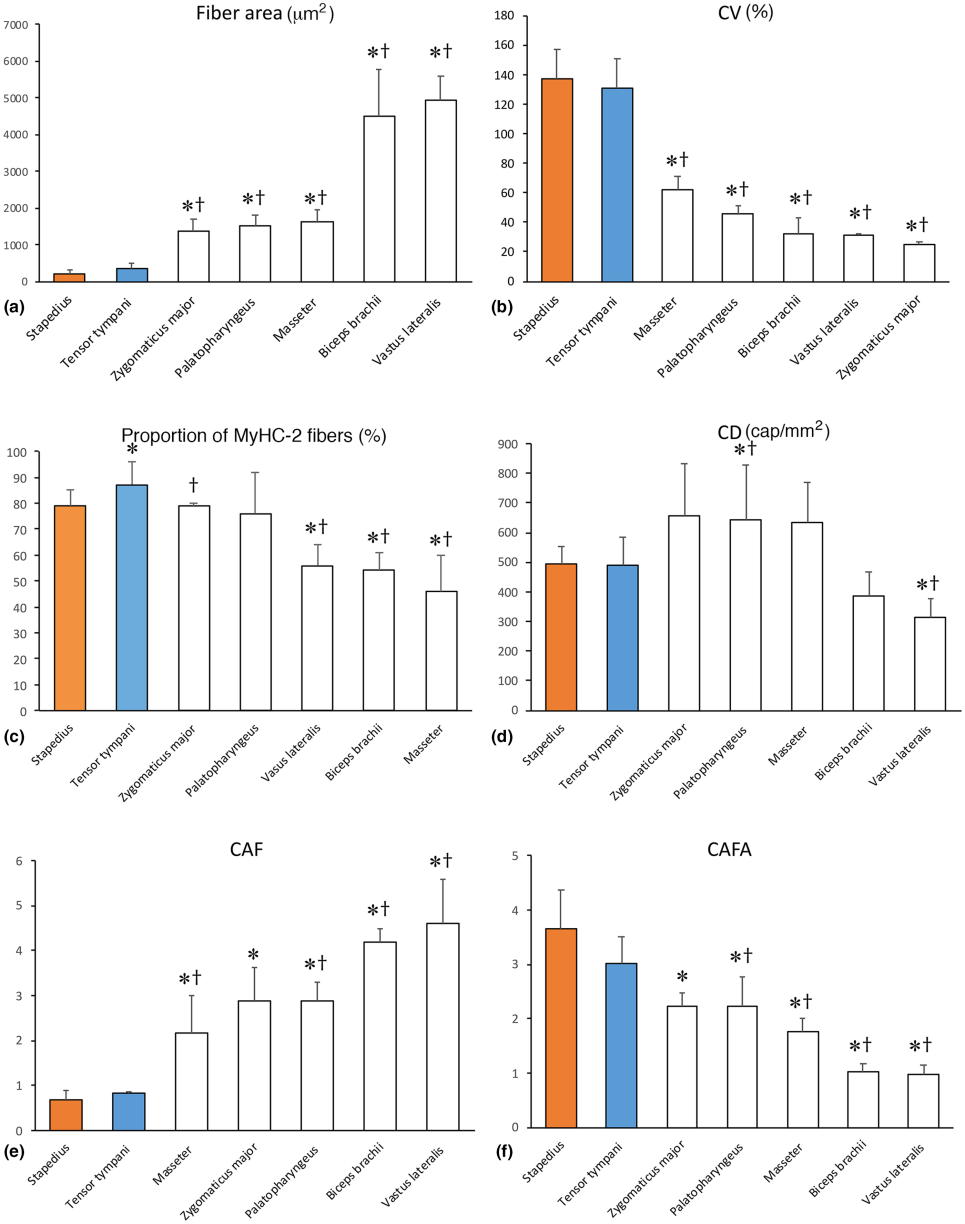
Comparison between human middle ear, cranial, and limb muscles. Bar graphs showing fiber area (a), variability in fiber area (CV) (b), percentage of MyHC‐2 fibers (c), capillary density (CD) (d), number of capillaries around fibers (CAF) (e), and capillaries around fibers relative to its cross‐sectional area (CAFA) (f) in the stapedius (orange) and tensor tympani (blue) muscles. For comparison, data for two different orofacial muscles, the zygomaticus major and palatopharyngeus, one masticatory muscle, the masseter, and two limb muscles, biceps brachii and musculus vastus lateralis, are included. Values are expressed as means and ± standard deviations. Statistical differences (*p* < 0.05) to the stapedius muscle are marked (*), and to the tensor tympani muscle are marked (†). Note the small fiber area (a), high variability in fiber size (b), high frequency of MyHC‐2 fibers (c), and a high number of capillaries per fiber area (f) in both middle ear muscles.

The mean fiber CSA was 220 ± 98 μm^2^, with no significant difference in fiber size between those fibers expressing slow MyHC‐1 (mean: 209 ± 115.8 μm^2^) and those expressing fast MyHC‐2 isoforms (mean: 223 ± 89 μm^2^). Hybrid fibers co‐expressing MyHC‐1/2A had the smallest mean fiber area (mean: 144 ± 60 μm^2^). The variability in fiber size (CV) was 137 ± 20% (Table 3; Figure 4).

**TABLE 3:** Muscle fiber area (μm^2^), the number of capillaries around fibers (CAF), and the number of capillaries around fibers relative to its cross‐sectional area (CAFA) of fibers expressing different MyHC isoforms in the stapedius and tensor tympani muscles.

Muscle	Fiber area (μm^2^)	CAF	CAFA
Stapedius			
MyHC‐1	209 ± 116	0.72 ± 0.12	4.13 ± 1.49
MyHC‐1/2A	144 ± 60*	0.34±0.23	2.01 ± 0.59
MyHC‐2A	225 ± 97	0.68 ± 0.03	3.66 ± 0.31
MyHC‐2A/2x	304 ± 133	0.55 ± 0.07	0.84 ± 0.84
MyHC‐2X	410^a^	^b^	^b^
Tensor tympani			
MyHC‐1	330 ± 187	0.99 ± 0.22	3.03 ± 1.45
MyHC‐1/2A	313 ± 157*	1.02 ± 0.36	2.88 ± 0.78
MyHC‐2A	358 ± 134	0.83 ± 0.23	3.08 ± 0.71
MyHC‐2A/2X	239 ± 140	0.79 ± 0.30	3.00 ± 0.55
MyHC‐2X	308 ± 209	0.59 ± 0.23	1.84 ± 0.59

*Note*: Data are presented as means ± standard deviations. Statistical differences between the muscles are marked * (*p* < 0.05).

^a^ Only observed in one case.

^b^ Too few fibers for a reliable calculation.

#### 3.2.4 Mitochondrial oxidative activity

Muscle fibers expressing slow MyHC‐1 generally exhibited strong NADH‐TR activity. The majority of fibers expressing fast MyHC‐2A or MyHC‐2X showed a moderate to strong NADH‐TR staining intensity, which was generally stronger than in the corresponding fibers in limb muscles (Figure 1).

#### 3.2.5 Capillarization

The average CD of the stapedius muscle was 494 ± 60 capillaries per mm^2^. The mean CAF was 0.68 ± 0.03, where those fibers expressing MyHC‐1 had a higher value than those expressing MyHC‐2 isoforms (CAF 0.72 ± 0.12 and 0.66 ± 0.03, respectively). When looking at the number of capillaries in relation to the fiber area (CAFA), the mean value was 3.66 ± 0.49, with the highest values for MyHC‐1 (CAFA 4.13 ± 1.49) and the lowest for MyHC‐2A/2X (CAFA 1.58 ± 0.84) (Table 3; Figures 3 and 4).

### 3.3 Musculus tensor tympani

#### 3.3.1 Frequency of muscle fiber phenotypes

Muscle fibers expressing fast MyHC‐2 isoforms predominated markedly in all samples (86 ± 9%), with fibers containing MyHC‐2A being the most frequent (mean 74 ± 17%). Fibers containing solely slow MyHC‐1 were observed in all the samples (mean: 14 ± 9%), while fibers containing fast MyHC‐2X were only found in three subjects (mean: 1 ± 2%). Seven of the nine muscle samples also displayed hybrid MyHC‐1/2A (5 ± 6%) and MyHC‐2A/2X (6 ± 10%) fibers (Table 2; Figure 1).

#### 3.3.2 Frequency of fibers expressing neonatal myosin

Muscle fibers co‐expressing MyHC‐neo were found in seven of the nine subjects (mean: 3.6 ± 3.3%) (Figure 2). MyHC‐neo was not co‐localized with any specific MyHC fiber type.

#### 3.3.3 Muscle fiber area and variability in fiber size

The mean fiber CSA was 360 ± 138 μm^2^. No differences in fiber size were found between fibers expressing MyHC‐1 and fibers expressing MyHC‐2 isoforms (mean 330 ± 187 vs. 358 ± 134 μm^2^). Muscle fibers containing MyHC‐2A showed the largest mean fiber area (358 ± 134 μm^2^); fibers co‐expressing MyHC‐2A/2X and solely MyHC‐2X had the smallest mean fiber area (239 ± 140 and 308 ± 209 μm^2^, respectively). The CV for all fibers was 131 ± 20% (Table 3; Figure 4).

#### 3.3.4 Mitochondrial oxidative activity

The NADH‐TR staining activity in the tensor tympani fibers was in the same range as in the stapedius muscle. Whereas fibers containing MyHC‐1 generally showed strong staining activity, fibers expressing MyHC‐2A or MyHC2X displayed a moderate to strong NADH‐TR staining intensity, which generally was stronger than in limb muscles (Figure 1).

#### 3.3.5 Capillarization

The average CD for all tensor tympani muscles was 428 ± 92 capillaries per mm^2^. The mean CAF was 0.84 ± 0.24, where fibers expressing MyHC‐1 and MyHC‐1/2A showed the highest CAF values (0.99 ± 0.22 and 1.02 ± 0.36, respectively). The CAFA mean value was 3.03 ± 0.72, with approximately the same values for MyHC‐1 and MyHC‐2A fiber phenotypes (3.03 ± 1.45 and 3.08 ± 0.71, respectively). Myosin heavy chain‐2X fibers showed the lowest CAFA value (1.84 ± 0.59) (Table 3; Figures 3 and 4).

### 3.4 Myosin heavy chain isoforms revealed by gel electrophoresis

**FIGURE 5:**
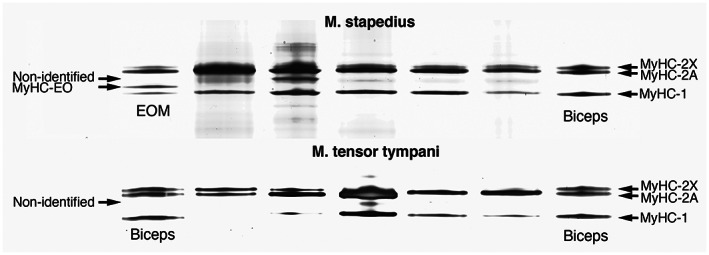
Myosin heavy chain (MyHC) isoforms in human middle ear muscles. Content of MyHC in 8% polyacrylamide‐SDS‐glycerol gels of five tensor tympani and five stapedius muscles. For comparison, one biceps brachii and one extraocular muscle (EOM) are included. The bands corresponding to slow MyHC‐1, fast MyHC‐2A, fast MyHC‐2X, and MyHC‐EO are indicated. Note the presence of a nonidentified band in one tensor tympani and five stapedius muscles.

The biochemical analysis revealed three major MyHC isoforms in the analyzed muscles: slow MyHC‐1, fast MyHC‐2A, and fast MyHC‐2X (Andersen & Schiaffino, 1997; Pereira Santana et al., 1997). Human MyHC‐2X was present in the slowest migrating band and MyHC‐1 in the fastest migrating band. Fast MyHC‐2A was the major isoform in both tensor tympani and stapedius muscles. Notably, the MyHC‐2X protein was generally present in a higher amount in the gels than derived from the antibody staining of muscle fibers. It was not possible to separate MyHC‐neo from the MyHC‐2A band in these gels, owing to either the similarities in mobility or the low amount of protein. A fourth band corresponding to MyHC‐EOM was seen in the EOMs just above slow MyHC‐1. In the stapedius muscle, the relative content (%) of MyHC isoforms was 24.5 ± 6.5% for MyHC‐1, 39.8 ± 19.0% for MyHC‐2A, and 22.9 ± 13.7% for MyHC‐2X. The corresponding values in the tensor tympani muscle were 15.7 ± 12.1% for MyHC‐1, 63.9 ± 21.2% for MyHC‐2A, and 18.8 ± 21.8% for MyHC‐2X. A nonidentified band migrating in between the fast MyHC‐2A and slow MyHC‐1 bands (Figure 5, arrows) was observed in all analyzed stapedius muscles (12.8 ± 7.4%) and three of the five analyzed tensor tympani muscles (1.6 ± 2.3%), but was not found in the biceps brachii or EOM muscles. The pattern of MyHC isoforms in the gels is shown in Figure 5.

### 3.5 Age and gender

When the data from the tensor tympani and stapedius muscles were divided according to subjects age, <60 years and ≥60 years, the mean proportion of hybrid MyHC‐1/2A and MyHC2A/2X fibers was significantly higher in the older group than in the younger group. No other statistical observations were found. Neither were any differences observed between genders.

### 3.6 Muscle fiber phenotypes in orofacial, jaw, and limb muscles

**FIGURE 6:**
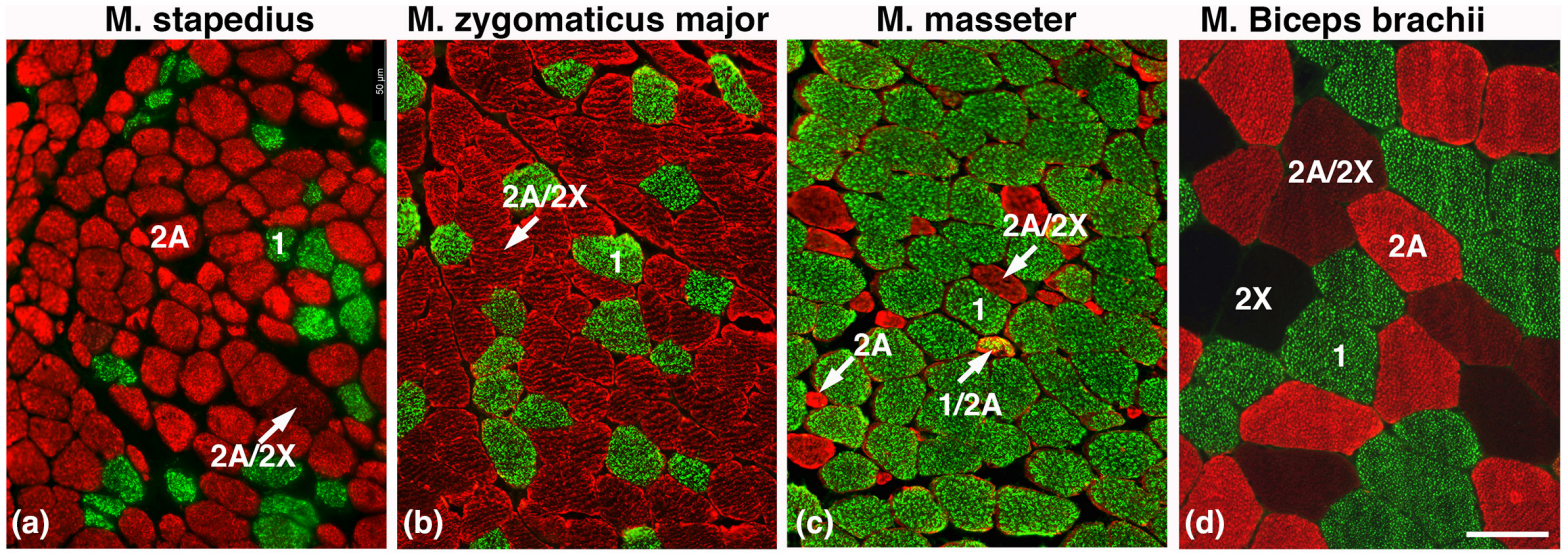
Comparison between middle ear, cranial, and limb muscles. Muscle cross‐sections from the stapedius (a), zygomaticus major (b), masseter (c), and biceps brachii (d) muscles stained for MyHC‐1 and MyHC‐2A. Muscle fibers expressing MyHC‐1 (green), MyHC‐1/2A (orange in c), MyHC‐2A (red), MyHC‐2A/2X (weak red), and MyHC‐2X (unstained) are marked. Note the higher proportion of MyHC‐2 fibers and the smaller fiber area in the stapedius muscle compared with the facial, jaw, and limb muscles. Scale bar 50 μm.

The limb and the orofacial and jaw muscles showed a MyHC fiber type composition as previously reported for these muscles (Pontén & Stål, 2007; Stål et al., 1987, 1990, 2003; Stål & Lindman, 2000; Staron et al., 2000). The biceps brachii and vastus lateralis muscles had an even distribution of relatively large, densely packed angulated fibers, about an equal proportion of which expressed MyHC‐1 and MyHC‐2 isoforms. The zygomaticus major muscle and palatopharyngeus were predominated by less densely packed, small‐sized rounded fibers containing MyHC‐2 isoforms. In contrast, the samples of masseter muscle were predominated by MyHC‐1 fibers and a relatively large proportion of small‐sized MyHC‐2X fibers (Table 2; Figure 6).

### 3.7 Comparison between middle ear, cranial, and limb muscles

The tensor tympani muscle had a significantly higher proportion of fibers containing MyHC‐2 isoforms compared to the stapedius muscle (86% vs. 79%, *p* = 0.04) (Figure 4). No significant differences were observed between the two middle ear muscles for fiber area and capillary parameters with the exception that the fiber area for MyHC‐1/2 fibers was significantly larger in the tensor tympani muscle (Figure 4; Table 3).

Both the stapedius and tensor tympani muscles generally shared more morphological similarities with the orofacial than with the jaw and limb muscles, i.e., the fibers were mainly composed of loosely packed and very small‐sized MyHC‐2 fibers with high NADH‐TR activity (Table 2; Figure 4). However, the middle ear muscles differed also from orofacial, jaw and limb muscles by having significantly higher variability in fiber size (CV) and more extensive capillarization per fiber area (CAFA) (Figure 4).

## 4 DISCUSSION

This is the first detailed study describing muscle characteristics of the human stapedius and tensor tympani muscles. The investigation revealed that the middle ear muscles have distinctive contractile, metabolic, and proprioceptive properties to meet the complex demands of auditory perception and the protection of inner ear structures. Although both muscles have many characteristics in common, they are also individually unique (Table 4).

**TABLE 4:** Muscle characteristics and known orofacial, pharyngeal, and middle ear factors influencing the activity of the human middle ear, facial, and limb muscles.

	M. stapedius	M. tensor tympani	M. zygomaticus major	M. biceps brachii
Nerve innervation	*N. facialis*	*N. trigeminus*	*N. facialis*	*Spinal nerve*
Mean muscle fiber area	220 µm^2^	360 µm^2^	1383 µm^2^	4508 µm^2^
Fiber size variability (CV)	Very high	Very high	High	Low
MyHC‐1/MyHC‐2 fiber types (%)	21/89	14/86	21/89	46/54
Fetal MyHC	Yes	Yes	No	No
Unidentified MyHC isoform	Yes	Yes	No	No
Contraction velocity	Fast	Fast	Fast	Moderate
Power per muscle area unit	High	High	High	Intermediate
Mitochondrial density in fibers	High to very high	High to very high	Moderate to high	Weak to high
Capillary density per fiber area	High	High	Intermediate	Intermediate to low
Endurance capacity	High	High	High	Intermediate
Nerve density in muscle	Very high	Very high	Moderate to high	Low
Muscle spindles	No	Yes	No	Yes
Bony attachments	Two ends	Two ends	One end	Two ends
Extracellular matrix	High	High	High	Low
Activated by				
Sound	Yes^1^			
Chewing		Yes^1^	Yes	
Swallowing		Yes^2^	Yes	
Eustachian tube opening		Yes^3^ ^a^		
Middle ear pressure changes		Yes^4^		
Skin stimulation face		Yes^2^		

*Note*: 1. (Mukerji et al., 2010), 2. (Bance et al., 2013), 3. (Sadé & Ar, 1997), 4. (Rodríguez‐Vázquez et al., 2016).

^a^ Collaborates with the tensor veli palatini muscle.

An interesting finding was that the stapedius and tensor tympani muscles had one of the highest proportions of fast‐contracting MyHC‐2 fiber types reported in any human muscle. Moreover, the gel electrophoresis showed a relatively high amount of MyHC‐2X protein, indicating that the muscles are even more powerful and fast‐contracting than revealed from the immunohistochemical analyzes. Further support for very rapid contractions is the observation of a protein band of unknown identity at the level of myosin in the gel‐electrophoretic separation. Smerdu and Cvetko have previously reported an unidentified band at the same level in human laryngeal muscles, which are considered to produce extremely fast movements (Smerdu & Cvetko, 2013). They showed that the laryngeal muscles contain the transcript for MyHC‐2B, the fastest MyHC isoform in mammals, but as in our study, they could not identify the protein in the gels using an antibody directed against MyHC‐2B. Given the short reflex time the ear muscles have in responding to loud sounds to protect the cochlea, it is reasonable to speculate that the stapedius muscle may contain an undetected, very fast protein isoform. Further investigations are required to ascertain whether human middle ear muscles contain a MyHC‐2B‐like isoform or another unique superfast MyHC isoform.

Another interesting observation was the presence of fibers co‐expressing developmental MyHC‐neo in the middle ear muscles. This MyHC isoform has been reported as a normal feature throughout adult stages in human masticatory, orofacial, and EOM muscles but not in limb muscles (Ciciliot & Schiaffino, 2010; Sartore et al., 1987; Stål et al., 1994; Stål & Lindman, 2000). Although the role of MyHC‐neo in the fibers is unclear, it has been speculated that neonatal myosin has low contraction velocity and is specialized in contractions against a low load (Schiaffino et al., 2015).

The relatively high proportion of hybrid fibers co‐expressing different MyHC isoforms indicates a functional continuum of physiological properties that give the middle ear muscles the ability for a fine gradual increase in force during movement (Larsson & Moss, 1993). Other factors predicting the ability of fine motor control are the fiber size and size of motor units. Muscles composed of small fibers and small motor units are well suited for force gradation because each motor unit develops relatively low forces. Even if the size of the motor units of the middle ear muscles is unknown, the small mean fiber area, the presence of hybrid fibers co‐expressing multiple MyHC isoforms, and the high content of nerve fascicles in the muscle cross‐sections suggest that both middle ear muscles are well suited to work with fine‐tuned motor control.

A significant determinant for oxygen delivery and the ability of the muscle cells to generate energy during aerobic conditions is the density and activity of myofibrillar mitochondria and the size of the capillary bed. Taking this into account, the relatively extensive capillarization and higher mitochondrial oxidative capacity of muscle fibers containing MyHC‐2A and MyHC‐2A/2X in the middle ear compared to limb muscles suggest that both tensor tympani and stapedius muscles have an unusually high capacity to work with fast and sustainable contractions under aerobic conditions. We have previously reported a similar high capillarization and oxidative capacity for MyHC‐2A fibers for some human orofacial muscles, highlighting a unique adaption of some fast muscle fibers in the cranial region (Granberg et al., 2010; Stål & Lindman, 2000).

The predominance of high oxidative fast‐contracting fibers with the ability for fine‐tuned movements supports the stapedius muscles widely accepted role as a protector of the cochlea in the inner ear against damaging sound levels. In excessive sound vibrations, the acoustic stapedius reflex contracts the muscle and tilts the stapes away from the fenestra vestibuli, which stiffens the ossicular chain. This acoustic reflex function explains why paralysis of the muscle causes hyperacusis (Rubini et al., 2020). The acoustic stapedius reflex is also reported to be involved in improved speech recognition at low‐frequency moderate noise levels (Aiken et al., 2013) as well as degraded speech recognition at high presentation levels (Shehorn et al., 2020). Further studies regarding the acoustic reflex feedback to the stapedius muscle are needed to confirm these theories.

Compared with the stapedius muscle, considerably less is known about the function of the tensor tympani muscle since no valid method of detecting its contraction exists. The muscle acts as an antagonist to the stapedius muscle and is proposed to form a functional unit with the levator veli palatini collaborating in the ventilation of the middle ear (Kierner et al., 2002). The muscle probably play an active part in middle ear pressure regulation and clearance since the tensor tympani is activated simultaneously with the levator veli palatini opening the Eustachian tube (Sadé & Ar, 1997). Even though contractions of the tensor tympani muscle have been audiometrically shown to result in low‐frequency mixed hearing loss (Wickens et al., 2017), there is evidence that the tensor tympani in humans is not activated by loud sounds from the environment and has non‐acoustic functions (Salomon & Starr, 1963). Several reports agree that the muscle is activated when exposed to self‐generated auditory stimuli such as swallowing, speaking, or chewing and nonauditory stimuli in the anticipation of loud sounds (Edmonson et al., 2022).

The tensor tympani tendon has been suggested to protect against outward displacement of the ossicular chain (Hüttenbrink, 1989). However, we have previously reported that the ligament by itself has very little effect in limiting the outward movement of the tympanic membrane and malleus (Rönnblom et al., 2021). In the same study, we found the combination of a negative pressure created by a finger being extracted from a wet ear canal and a simultaneous counteracting reflexive force by the tensor tympani muscle could cause an isolated malleus fracture with an intact tympanic membrane. Thus, the high proportions of fibers with fast, relatively powerful, and sustainable contractions in the human tensor tympani muscles, together with the presence of muscle spindles contributing to a modulated stretch reflex response, highlights its well‐suited role in protecting the ossicular chain and the inner ear from the excessive outward movement of the tympanic membrane and the ossicular chain.

The presence of muscle spindles in the tensor tympani muscle, but the lack of typical spindles in the stapedius muscle, highlights the different roles of these muscles. As the muscle spindle is a sensory receptor that primarily transmits changes in the length and speed to the central nervous system, which in turn coordinates adequate motor neuron activity to resist muscle stretching, the tensor tympani muscles seem better adapted for flexible sensory performance than the stapedius muscle. The difference in proprioceptive control of the muscles may be attributed to the fact that the stapedius muscle is involved in reflex movements that rapidly pull the stapes sideways to protect the inner ear from loud noises while the tensor tympani muscle pulls the tympanic membrane rapidly inward but in a more varied and fine‐tuned movement pattern.

We conclude that the human middle ear muscles have a highly specialized muscle morphology and fiber phenotype composition with specific contractile, metabolic, and proprioceptive properties that have more similarities with human orofacial than with jaw and limb muscles. The high proportion of fast‐contracting MyHC‐2 fibers with pronounced mitochondrial oxidative capacity, the relatively high presence of hybrid fibers, and the extensive capillarization and density of nerve fascicles in both the stapedius and tensor tympani muscles show that these muscles are adapted to perform rapid fine‐tuned contractions with relatively high endurance. However, despite these similarities, they have different functions in the middle ear. The stapedius muscle, which lacks ordinary muscle spindles, has a muscle fiber composition adapted to act rapidly in a fine‐tuned manner stiffening the ossicular chain through the acoustic reflex. In contrast, the human tensor tympani muscle which contain muscle spindles, perform various non‐acoustic functions in a fast and precise fashion and assists in ventilation of the middle ear. Stretch reflexes triggered by muscle spindles most likely prevent excessive outward movements of the tympanic membrane avoiding disruption of the ossicular chain or damage to the inner ear.

## AUTHOR CONTRIBUTIONS

Per Stål and Lars‐Eric Thornell were responsible for the conceptualization and collection of autopsies. Per Stål, Anton Rönnblom, and Krister Tano performed experiments and collected data. Anton Rönnblom and Farhan Shah were responsible for the statistical analysis. All authors contributed to the evaluation of data, writing, and editing of the article. Per Stål was responsible for the supervision and final editing. All authors have read and approved the final article.

## CONFLICT OF INTEREST STATEMENT

The authors declare that there is no conflict of interest.

## Data Availability

The data that support the findings of this study are available from the corresponding author upon reasonable request.
